# The build-up of osmotic stress responses within the growing root apex using kinematics and RNA-sequencing

**DOI:** 10.1093/jxb/erw350

**Published:** 2016-10-04

**Authors:** Mathilde Royer, David Cohen, Nathalie Aubry, Vera Vendramin, Simone Scalabrin, Federica Cattonaro, Marie-Béatrice Bogeat-Triboulot, Irène Hummel

**Affiliations:** ^1^UMR EEF, INRA, Université de Lorraine, 54280 Champenoux, France; ^2^IGA Technology Services, I-33100 Udine, Italy

**Keywords:** Division, elongation, hormonal signaling, osmotic stress, *Populus nigra*, root apical meristem, root growth, transcriptome

## Abstract

Molecular regulation of growth must include spatial and temporal coupling of cell production and cell expansion. The underlying mechanisms, especially under environmental challenge, remain obscure. Spatial patterns of cell processes make the root apex well suited to deciphering stress signaling pathways, and to investigating both processes. Kinematics and RNA-sequencing were used to analyze the immediate growth response of hydroponically grown *Populus nigra* cuttings submitted to osmotic stress. About 7400 genes and unannotated transcriptionally active regions were differentially expressed between the division and elongation zones. Following the onset of stress, growth decreased sharply, probably due to mechanical effects, before recovering partially. Stress impaired cell expansion over the apex, progressively shortened the elongation zone, and reduced the cell production rate. Changes in gene expression revealed that growth reduction was mediated by a shift in hormone homeostasis. Osmotic stress rapidly elicited auxin, ethylene, and abscisic acid. When growth restabilized, transcriptome remodeling became complex and zone specific, with the deployment of hormone signaling cascades, transcriptional regulators, and stress-responsive genes. Most transcriptional regulations fit growth reduction, but stress also promoted expression of some growth effectors, including *aquaporins* and *expansins*. Together, osmotic stress interfered with growth by activating regulatory proteins rather than by repressing the machinery of expansive growth.

## Highlight

Osmotic stress rapidly induces strong transcriptome responses within the root apex and interferes with growth through a deep remodeling of hormonal status rather than by repressing the machinery of expansive growth.

## Introduction

Root growth is responsive to changes in the environment (Walter *et al.*, 2009). The biophysics of root growth has been studied in response to different environmental cues, such as low temperature (Pritchard *et al.*, 1990), carbon (Muller *et al.*, 1998), or water (Sharp *et al.*, 1988; Spollen *et al.*, 2008). Following a sudden change in water availability, growth shows a two-phased response: an acute response followed by a new, usually lower, steady-state growth rate (Skirycz and Inzé, 2010). Most studies have focused on comparing root growth in the two successive steady states (before stress versus the new steady growth rate, e.g. Spollen *et al.*, 2008), while the responses occurring during the transition period remain largely undocumented. Pritchard *et al.* (1991) and Frensch and Hsiao (1994) showed that cell turgor pressure decreased very rapidly following the onset of osmotic stress, stopping growth, but was restored in growing cells in at most a couple of hours. Growth was then only partially restored, indicating that molecular and physiological rewiring had taken place in addition to physical responses.

Aiming to discover key regulators of drought acclimation, drought-induced molecular responses have been studied extensively (Lawlor, 2013). Altering the expression of regulators of drought responses has enhanced drought tolerance but often at the cost of growth, suggesting that the molecular pathways controlling growth and drought response are intertwined. Some studies have specifically focused on transcriptome remodeling in growing organs such as the root apex (e.g. Cohen *et al.*, 2010). Others have gone further, depicting drought-induced transcriptional changes in differentially responding sections along the root apex (Poroyko *et al.*, 2007; Opitz *et al.*, 2016). Indeed, since root growth involves both cell proliferation and cell expansion, which occur sequentially along the root apex, harmonious and sustainable growth requires that the activities of these two processes are coupled (Silk, 1992; Baskin, 2013). Sensitivity of cell elongation to water deficit has been shown in several species using kinematics (Sharp *et al.*, 1988; Triboulot *et al.*, 1995). The importance of apical meristem activity in determining growth rate has been highlighted (Beemster and Baskin, 1998), and its sensitivity to water deficit has been shown in maize and poplar roots (Fraser *et al.*, 1990; Sacks *et al.*, 1997; Bizet *et al.*, 2015*b*). The balance between proliferation and elongation processes, as well as their underlying molecular control, is a current research focus (Tardieu *et al.*, 2011; Baskin, 2013; De Vos *et al.*, 2014). A gene expression map at high spatial resolution has led to better knowledge of the transcriptional circuits linked to root development in *Arabidopsis thaliana* (Birnbaum *et al.*, 2003; Brady *et al.*, 2007; Iyer-Pascuzzi *et al.*, 2009). Takatsuka and Umeda (2014) pointed out the importance of considering the roots of plant species other than Arabidopsis in order to gain further insights into the networks involved in growth regulation. RNA-sequencing now allows a holistic view of the root transcriptome in any species. Relative to the small roots of Arabidopsis, the large adventious roots of poplar cuttings are easier to dissect and allow continuous monitoring of growth.

To better understand how proliferation and elongation responses are coupled in a growing organ, attention must be paid to the dynamics of environmentally induced changes in growth rate (Silk, 1992; Sharp *et al.*, 2004; Beemster *et al.*, 2005). In this study, we analyzed the immediate growth response to osmotic stress within the root apex of poplar, using kinematics and RNA-sequencing. We addressed the question of the timing of transcriptome remodeling (by sampling roots both 30min and 3h after stress onset) considering both proliferation and elongation processes (by analyzing the transcriptome within the division zone and the elongation zone separately). In the whole root system of Arabidopsis, gene expression was rapidly altered after osmotic stress onset, triggering a continuous response which increased with time (Kilian *et al.*, 2007). We thus hypothesized that the zone specificity of the responses would build up over these early steps. We aimed to assess the molecular events involved in the establishment of growth inhibition.

Osmotic stress reduced both cell expansion and cell production in *Populus* root. As early as 30min after stress onset, signaling pathways were activated, with the earliest steps involving hormone metabolism in particular. Our work highlights the canonical function of abscisic acid (ABA) in the growth response to osmotic stress, as well as its interplay with other hormones. Transcriptome rewiring became more complex as growth reached a new steady state. Distinct groups of genes were identified in each zone. Comparison with transcriptome responses to drought in soil suggested that some of these molecular events were not transient, but steadily activated for a long-term acclimation.

## Materials and methods

### Plant material, growth conditions, and stress application

*Populus nigra* cuttings (genotype 6J29) were grown as in Bizet *et al.* (2015*b*). The hydroponic tanks were randomly assigned to control or stress conditions. Osmotic stress was applied by replacing the nutrient solution with one supplemented with 160g l^−1^ polyethylene glycol (PEG 4000, Merck Chemicals, Darmstadt, Germany), generating an osmotic potential of −0.35MPa (Wescor 5500, Logan, UT, USA), and without lowering the oxygen level (oxymeter HQ40D, Hach Lange, Noisy-le-grand, France) (Supplementary Fig. S1 at *JXB* online). In controls, the nutrient solution was replaced by fresh solution. Solutions were fully replaced in <10min.

Control and osmotic stress treatments were performed in parallel, with two batches of eight cuttings per treatment. The experiment was run twice, independently. Apices of roots longer than 2cm were collected 0.5h and 3h after the change of nutrient solution and stored in RNAlater^®^ (Ambion, Austin, TX, USA).

### Root growth monitoring, growth parameter determination, and microscopy

Once a root was longer than 2cm, the cutting was transferred to a transparent tank filled with circulating nutrient solution. Root growth was monitored in the dark under near-infrared illumination as in Bizet *et al.* (2015*b*). In this device, the PEG-added solution fully replaced the control solution in 3min, without any manipulation of the root or pause in the growth monitoring. Images were taken every 6min (Supplementary Fig. S2A). Raw velocity profiles were obtained by particle image velocimetry, using Kineroot (Basu *et al.*, 2007). The velocity profile along the root apex was interpolated with a cubic smoothing spline (smooth.spline, spar 0.5, R Development Core Team, 2014). The elemental elongation rate (EER) profile was calculated by spatially differentiating the velocity profile (Erickson, 1976). Root growth rate was determined as the maximum velocity (Supplementary Fig. S2B). Growth zone length was measured as the distance between the quiescent center and the position where EER fell below 3%. EER_max_ was defined as the maximum value of EER (Supplementary Fig. S2C). The growth zone includes the so-called division zone (DZ), where cells divide and slowly expand, and the elongation zone (EZ) where cells rapidly elongate. The length of the DZ was determined using infrared brightness as in Bizet *et al.* (2015*b*). Briefly, the drop of brightness below 70% of its maximum, probably due to lower cell wall density, corresponded to the end of DZ (Supplementary Fig. S2D). EZ length was calculated as growth zone length minus DZ length.

Following growth monitoring, root apices were fixed and sectioned longitudinally to determine the cell length profile (Bizet *et al.*, 2015*b*; Supplementary Fig. S2E). Cell production rate was computed as the ratio between velocity at the end of the DZ and mean cell length in the apical meristem.

### RNA isolation, library preparation, and Hiseq 2500 sequencing

Root apices were dissected under a microscope in RNAlater^®^. After removal of the 0.5mm apical part of the root (corresponding to the root cap), two consecutive segments were collected: (i) a 1.5mm long segment corresponding to the DZ; and (ii) a 4.5mm long segment corresponding to the EZ. Four replicates were built by pooling eight root segments per sampling unit (zone×treatment×time). Root segments in DZ and EZ were paired-distributed (DZ and EZ libraries with the same index were constructed from the same pool of roots). The two experiments were randomly represented among replicates.

RNA was extracted using a Spectrum Plant Total RNA kit (Sigma, St Louis, MO, USA) with a DNAse I treatment. Absence of genomic DNA was confirmed by PCR with intron-flanking primers. RNA quality was assessed using an Experion RNA StdSens Analysis kit (Bio-Rad, Hercules, CA, USA).

Real-time PCRs were performed with 250ng of RNA, as described in Bizet *et al.* (2015*a*). Amplicons were sequenced (Supplementary Fig. S3).

RNA were checked on Caliper GX (PerkinElmer, Waltham, MA, USA). Libraries were prepared with 1.5 µg of RNA using a ‘TruSeq Stranded mRNA Sample Prep kit’ (Illumina, San Diego, CA, USA) with an 8-fold multiplexing level. mRNA was fragmented for 3min at 94 °C. Purification was performed using 0.6X Agencourt AMPure XP beads (Beckman Coulter, Villepinte, France). Libraries were quantified using the Qubit 2.0 Fluorometer (Invitrogen, Carlsbad, CA, USA) and quality tested by Agilent 2100 Bioanalyzer High Sensitivity (Agilent Technologies, Santa Clara, CA, USA). RIN values of samples ranged from 7.3 to 9.4 (median=9). Libraries were processed with Illumina cBot for cluster generation on the flow cell, and sequenced in paired-end mode on HiSeq2500 (Illumina). Raw data processing was performed using CASAVA v1.8.2 of the Illumina pipeline for both format conversion and de-multiplexing.

Reads were deposited in the Sequence Read Archive (SRA) under study accession number SRP067564.

### RNA-sequencing processing

Quality filtering and trimming of paired-end reads were performed using the erne-filter command (Erne v1.3, default parameters except --min-size=70; Del Fabbro *et al.*, 2013). For each sample, reads were mapped to the *Populus trichocarpa* genome sequence (unmasked v3.0 Phytozome v10) using TopHat2 v2.12 (Trapnell *et al.*, 2009; D. Kim *et al.*, 2013). The *P. trichocarpa* genome was indexed with the ‘bowtie-build’ command from the genome sequence fasta file. TopHat2 default parameters were used except: -r 235, --mate-std-dev 55, -g 10, --read-mismatche 10, --read-edit-dist 10, and --read-gap-length 10. Gene features were provided with the –G option (Ptrichocarpa_210_v3.0.gene_exons.gff3 file). Reads that mapped once were extracted from BAM files (accepted_hits.bam, TopHat2 output) using the NH:i:1 tag as described by Loraine *et al.* (2013). Further analyses were performed on single-mapping reads. A reference annotation-based transcript assembly was constructed (RABT; Roberts *et al.*, 2011). Assemblies were performed using Cufflinks v2.2.1 (Trapnell *et al.*, 2010), which was supplied with the Ptrichocarpa_210_v3.0.gene_exons.gff3 file. A merged.gtf file was created from the 32 transcripts.gtf output files using Cuffmerge.

Based on their mapping, the 41 226 loci were assigned to three categories: unique predicted gene, putative uTAR (unannotated transcriptionally active regions), or chimera (spanning over several predicted genes or tandem repeats). Ambiguous contigs due to assembly artifacts were removed.

### Expression analysis and detection of differentially expressed genes

Cuffdiff2, supplied with the merged.gtf file, was used with default parameters (Trapnell *et al.*, 2012). For each locus, the number of single-mapping reads gave access to a normalized count-based expression that was used to perform a hierarchical clustering of the 32 libraries (Euclidean distance using R package Stats). Mean and normalized expression values were computed as FPKM (fragments per kilobase of transcript per million mapped reads). Eight pairwise comparisons were designed on the basis of their biological relevance (differential expression between zones: EZ-CTL-0.5h versus DZ-CTL-0.5h, EZ-CTL-3h versus DZ-CTL-3h, EZ-PEG-0.5h versus DZ-PEG-0.5h, and EZ-PEG-3h versus DZ-PEG-3h; stress response: DZ-PEG-0.5h versus DZ-CTL-0.5h, DZ-PEG-3h versus DZ-CTL-3h, EZ-PEG-0.5h versus EZ-CTL-0.5h, and EZ-PEG-3h versus EZ-CTL-3h). A locus was denoted ‘expressed’ or ‘not expressed’ from its status in Cuffdiff2. No significant expression was found for 13 933 loci (call ‘Notest’), and checked on normalized count and FPKM. Differential gene expression was expressed as the Log2 of the fold change (FC). Differentially expressed genes (DEGs) were identified by Cuffdiff2 (Student *t*-test, *P*-value <0.05) after correction for multiple testing [false discovery rate (FDR), *P*-value <0.05] and applying the threshold (|Log2(FC)|≥2).

### Identification, *in silico* validation, and functional annotation of uTARs

The *P. trichocarpa* genomic sequence at the uTAR location was used as the query for a homology search in plant databases. BLASTN was performed against the expressed sequence tag (EST) database with default parameters and 10^–5^ as the maximal e-value (NCBI, http://www.ncbi.nlm.nih.gov), and the best matched poplar EST was reverse-blasted on the *P. trichocarpa* genome v3.0 (Suppplementary Table S3). NCBI BLAST+ executable (v2.2.8) was used on a local platform against the Non-Redundant Protein Sequence database (April 2014). The command line ‘blastx’ was executed with default parameters except that the e-value threshold was set to 1. BLAST results and the JBrowse interface were used to discard uTARs mapped on repeats.

### Gene annotation, ontology, and enrichment analysis

Gene ontology (GO) assignment, GO-Slim summarization, and enrichment analysis were performed using Blast2Go (v2.8; Conesa *et al.*, 2005). The 41 335 *P. trichocarpa* primary transcripts were blasted against *A. thaliana* (‘blastx’, default parameters, except e-value threshold=0.01). Only the best hit was imported, linking poplar gene models to the GO identifiers of their closest Arabidopsis orthologs. GO was summarized as plant GO-Slim terms. Enrichment analysis consisted of a Fisher’s exact test combined with an FDR correction for multiple testing. A corrected *P*-value of 0.05 was used to identify enriched GO terms.

### Arabidopsis microarrays

Three Arabidopsis studies were selected for a pair-wise comparison of DZ and EZ (Birnbaum *et al.*, 2003; De Rybel *et al.*, 2010; Tsukagoshi *et al.*, 2010). Affymetrix ATH1 Arrays were normalized using R (gcrma 2.40, default parameters; Irizarry *et al.*, 2003). Differential expression was computed for Columbia wild-type as Log2(FC), moderated *t*-tests implemented in the eBayes function (limma 3.24.12; Smyth, 2004), and FDR corrections for multiple testing were employed. A zone-preferred expression [corrected *P*-value <0.05, Log2(FC)≥2] was detected for 1173, 2972, and 4349 AGI in GSE21876, E-MEXP-2912, and GSE5749, respectively.

## Results

### The dynamics of root growth in response to osmotic stress

**Fig. 1.**
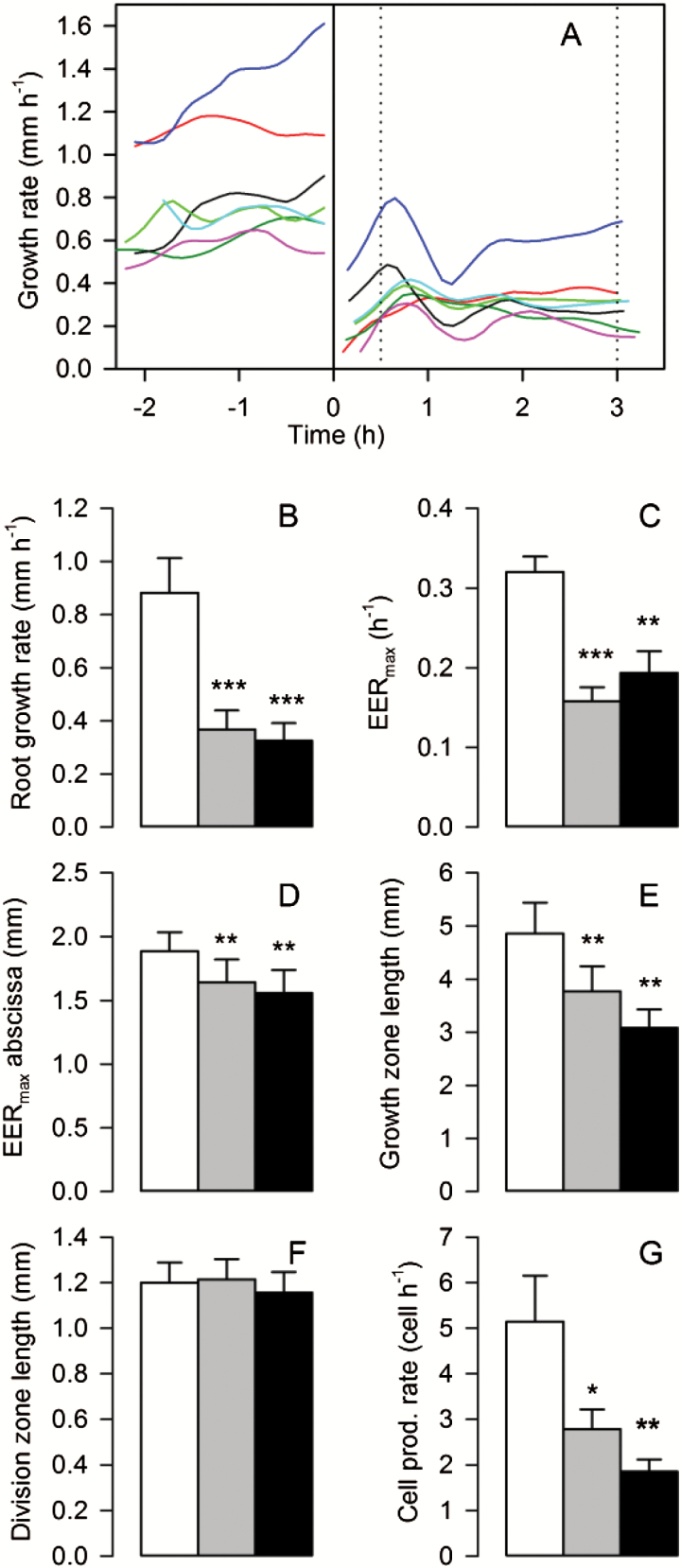
Root growth response to osmotic stress. (A) Individual root growth rate versus time. The solid line indicates the time of stress onset, the dotted lines the harvest time points. (B–G) Growth parameters measured before stress, and after 0.5h and 3h under stress (white, gray, and black, respectively): (B) root growth rate, (C) maximal elemental elongation rate (EER_max_), (D) EER_max_ abscissa, (E) growth zone length, (F) division zone length, and (G) cell production rate. Mean ±SE, *n*=7, asterisks denote paired *t*-tests *P*-value (***<0.001, **<0.01, and *<0.05) after ANOVA.

As the growth of poplar root is relatively sensitive to osmotic stress, a moderate osmotic stress (−0.35MPa) was applied by adding PEG to the nutrient solution. The growth response to osmotic stress of poplar roots was determined under infrared illumination. Our experimental set-up enabled the growth of individual roots to be monitored without any root manipulation. Before stress, the mean growth rate was 0.83mm h^−1^ but with a large range (Fig. 1A). Immediately following the onset of stress, root contraction and movements prevented focus and growth monitoring for a few minutes, probably due to changes in cell turgor. After 10min under stress, root growth was strongly reduced but was already recovering. During this transition period, all roots showed a common pattern of growth rate, with smooth oscillations passing through a maximum within 45min of stress application. Growth rate reached a new steady state after 2h under stress.

Kinematic analyses were performed during the transition period (after 0.5h under stress) and during the steady states (before stress and after 3h under stress). Osmotic stress significantly decreased the root growth rate to the same extent at both time points (Fig. 1B). The maximal elemental elongation rate (EER_max_) was decreased by 50%, and its position on the root axis was shifted towards the quiescent center (Fig. 1C, D). The growth zone was reduced according to stress duration (Fig. 1E). In contrast, the length of the division zone remained unchanged throughout the experiment (Fig. 1F). The rate of cell production by the meristem was also reduced (Fig. 1G). Since osmotic stress did not impact cell length within the division zone (Supplementary Fig. S2F), the reduced cell production rate reflected a reduced cell division rate. Taken together, osmotic stress reduced both cell processes (production and expansion), as well as the length of the elongation zone, but not that of the division zone.

### Quality of RNA-sequencing raw data

**Fig. 2.**
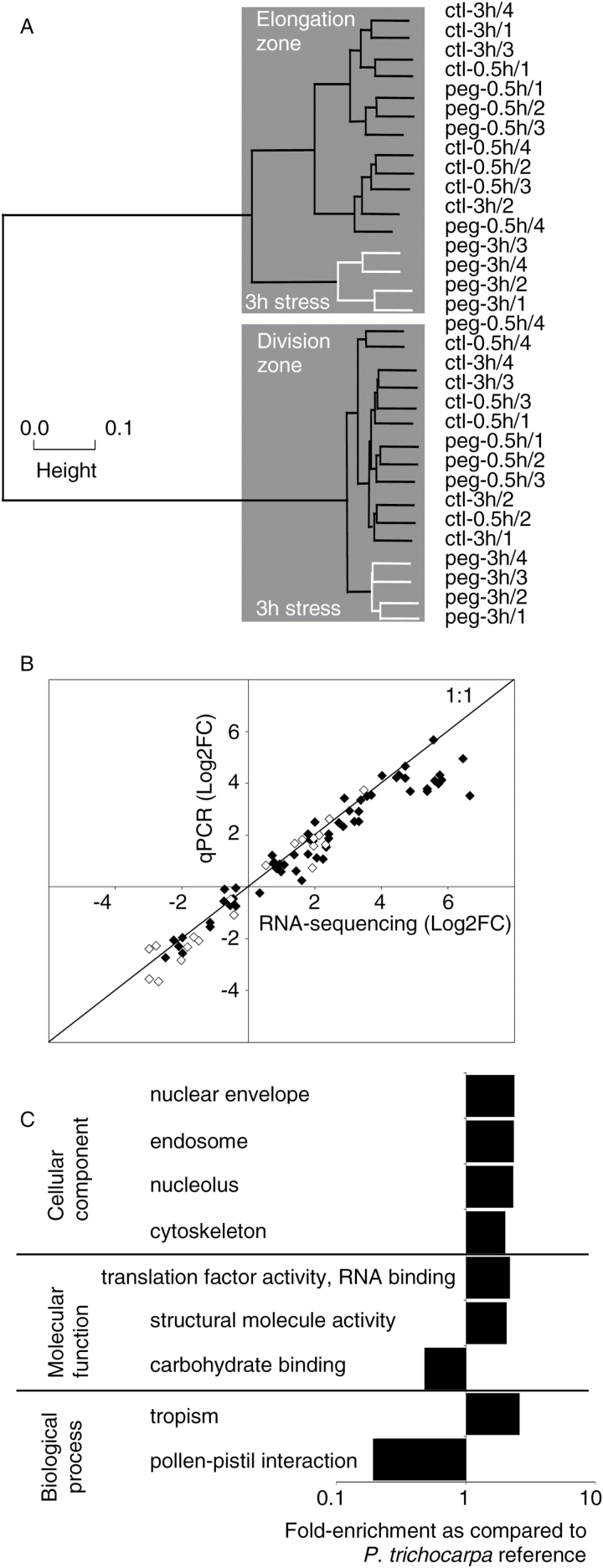
The root apex transcriptome. (A) Hierarchical clustering performed on normalized count-based expression. Gray boxes highlight the main clusters gathering libraries from the division zone and from the elongation zone. The second-order clustering (white) highlights 3h long osmotic stress libraries. (B) Fold changes in gene expression assessed by RNA-sequencing and qPCR. Black and white symbols correspond to annotated genes and uTARs, respectively (Supplementary Fig. S3). (C) Highest GO enrichments in the root apex transcriptome as compared with the *P. trichocarpa* genome (|Fold-change enrichment| ≥2, corrected *P*-value <0.05, Blast2GO;  Supplementary Table S2).

The dynamics of transcriptome remodeling under osmotic stress were investigated in the DZ and the EZ using 32 cDNA libraries. RNA-sequencing was performed using Hiseq2500 technology, generating ~30 million pair-end reads of 100bp per library (Supplementary Table S1). Following quality filtering, reads were mapped onto the *P. trichocarpa* genome using a reference annotation-based transcript assembly. Only reads that mapped exactly once onto the genome were considered (~95% of mapped reads; Supplementary Table S1). Overlapping reads per locus were counted and normalized using Cuffdiff2 (~13 million counts per library). Transcriptomes were strongly structured according to zone (Fig. 2A). Within the DZ and EZ clusters, the libraries corresponding to roots collected after 3h under stress were gathered together, indicating not only the impact of this treatment on the transcriptome but also its reproducibility. The remaining libraries were close, indicating a temporal stability of gene expression under control conditions and only minor changes during the transition period. Normalized expression was computed per sampling unit (zone×treatment×time) as the mean of four independent biological replicates and expressed in FPKM. RNA-sequencing results were confirmed by qPCR for 23 loci, with both methods being highly consistent (*y*=0.85*x*, *R*^2^=0.92, *n*=132; Fig. 2B; Supplementary Fig. S3).

### The root apex transcriptome

The transcriptome of the *P. nigra* root apex consisted of 25 262 expressed genes, covering ~62% of the 41 335 loci containing protein-coding transcripts of the *P. trichocarpa* v3 genome. Using Blast2go, 92% of these transcripts were associated with a GO. As compared with the *P. trichocarpa* genome, the root apex transcriptome was significantly enriched in 96 GOs while 41 GOs were significantly under-represented (Fig. 2C; Supplementary Table S2A). For cellular component, the most significant enrichments were ‘nuclear envelope’, ‘endosome’, ‘nucleolus’, and ‘cytoskeleton’, while ‘extracellular space’ was the most strongly depleted term. In terms of molecular function, ‘structural molecule activity’ and ‘translation factor activity, RNA binding’ were over-represented, while ‘carbohydrate binding’ was under-represented. The main enrichments for biological process were ‘tropism’ and ‘translation’.

RNA-sequencing revealed some putative novel transcribed regions (uTARs), which were inspected manually. Based on homology searches and JBrowse inspection, 258 uTARs were cross-validated. They were not only expressed in *P. nigra* libraries and mapped onto the *P. trichocarpa* genome but were also supported by a poplar mRNA and/or matched a predicted protein motif (Supplementary Table S3). About 20% of the cross-validated uTARs overlapped a predicted transcript in the previous version of the *P. trichocarpa* genome.

### Expression patterns within the growth zone

A large compendium of genes exhibited differential expression in the DZ and EZ under at least one condition (time×treatment) (Supplementary Table S4; 7341 genes), and, for >70% of them, consistently under the four conditions (Table 1).

**Table 1.** Number of differentially expressed genes

Differential expression^*a*^ in the division zone (DZ) and the elongation zone (EZ)	Under at least one condition (time×treatment)	7341 DEGs
	Under the four conditions (time×treatment)	5152 DEGs
	DZ-preferred expression	1953 DEGs
	EZ-preferred expression	3199 DEGs
Differential expression^*a*^ in response to stress	Under at least one comparison (zone×time)	1162 DEGs
	In the DZ In the EZ	216 DEGs 393 DEGs

^*a*^ Student *t*-test *P*-value <0.05; FDR *P*-value <0.05 and threshold, |Log2(FC)| ≥2.

**Fig. 3.**
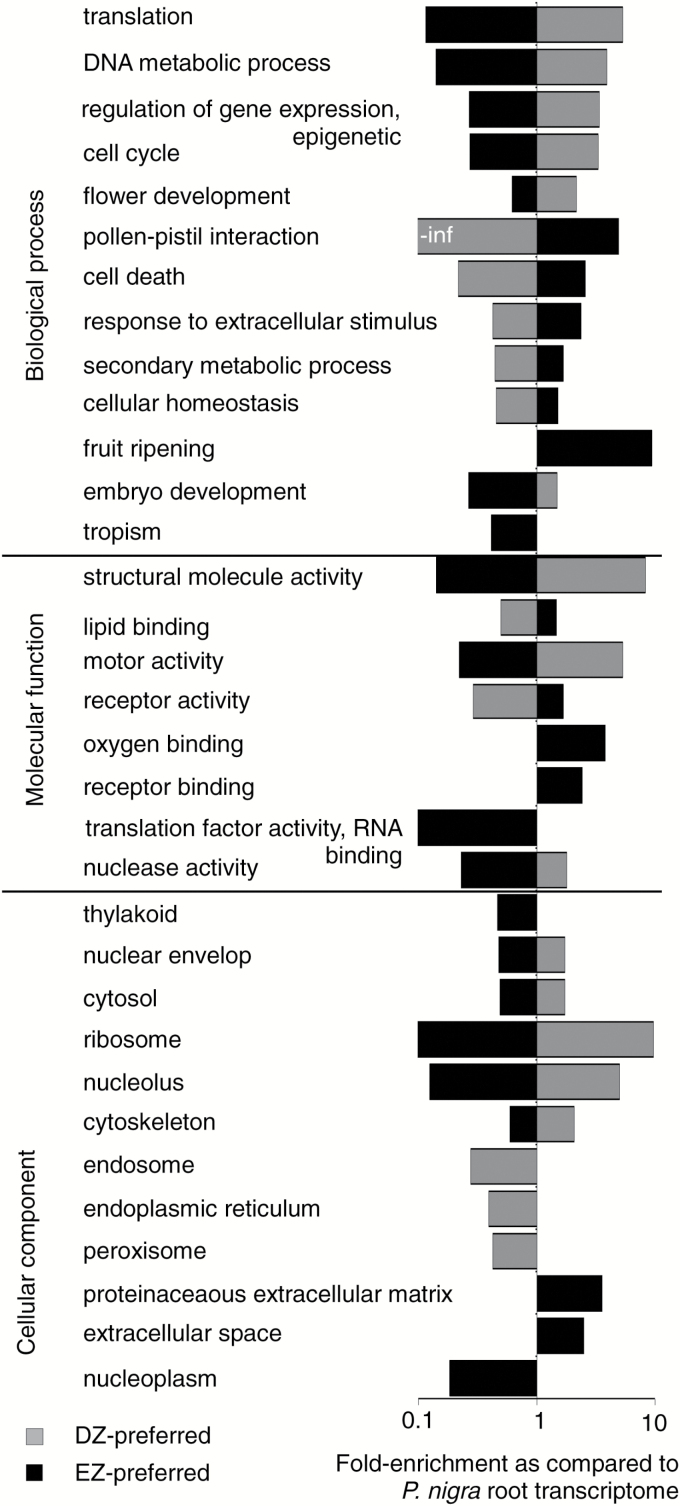
Transcriptomes of the division and elongation zone. Highest GO enrichments among zone-preferred expressed *Populus* genes in the division zone (gray bars) and in the elongation zone (black bars) as compared with *P. nigra* root transcriptome (|fold change enrichment| >2, corrected *P*-value <0.05; Supplementary Table S2).

A total of 1953 genes and 18 uTARs were preferentially expressed in the DZ (‘DZ-preferred’; Supplementary Tables S3, S4). Among them, 910 genes and five uTARs exhibited at least 4-fold higher expression in the DZ than in the EZ, regardless of growth conditions. Comparing the DZ-preferred genes with the root apex transcriptome revealed significant functional enrichments (Fig. 3; Supplementary Table S2B). As expected for actively dividing cells, the most enriched GO terms were ‘translation’, ‘DNA metabolic process’, ‘regulation of gene expression’, and ‘cell cycle’, and encompassed a large set of genes associated with ‘ribosome’, ‘nucleolus’, and ‘cytoskeleton’. On the other hand, 3199 genes and 24 uTARs were expressed preferentially in the EZ, including 2011 genes and 14 uTARs showing at least 4-fold higher expression in the EZ than in the DZ (‘EZ-preferred’; Supplementary Tables S3, S4). The most enriched GO term was ‘fruit ripening’, gathering genes involved in the synthesis of ethylene (Fig. 3; Supplementary Table S2B). For cellular component, ‘proteinaceous extracellular matrix’ and ‘extracellular space’ were over-represented.

With the exception of ‘cell wall’ (Supplementary Table S2B), functional enrichments were zone specific. For instance, ‘receptor binding’ or ‘oxygen binding’ were over-represented among the EZ-preferred genes. Similarly ‘flower development’ was over-represented among the DZ-preferred genes, due to the association of this ontology with genes related to cell cycle and chromatin structure. Notably, most of the GO terms over-represented among DZ-preferred genes were significantly depleted among EZ-preferred genes, and vice versa. Such over- and under-representations of functional groups in the two core sets corroborated the proper sampling of the DZ and EZ during root dissection.

A cross-species comparison was carried out using the AGI of the closest *Arabidopsis* homolog. Agreeing with the well-known amplification of gene number in the *Populus* genome, the 7341 *Populus* genes that showed a differential expression in the DZ and EZ (Table 1) were related to 4752 AGI (Supplementary Table S4). Among them, 1730 AGI exhibited a zone-preferred expression in the Arabidopsis root apex, thus consolidating the expression patterns of 2410 *Populus* genes. Such a strong overlap was unexpected, especially considering the highly duplicated poplar genome.

The overview of gene functions confirmed that genes related to cell division and cell elongation were expressed in the expected zones (Supplementary Table S4). Most *cyclin* and *cyclin-dependent kinase* genes showed a DZ-preferred expression, as did the 86 *Populus* genes for which the closest Arabidopsis gene was expressed preferentially in the quiescent center (Nawy *et al.*, 2005). Genes related to cell wall modification (such as *xyloglucan endotransglucosylase / hydrolase or expansin*) exhibited mostly EZ-preferred expression. *WRKY* transcription factor genes were expressed preferentially in the EZ. Concerning the 41 *aquaporins* expressed in the root apex, transcripts accumulated preferentially in the EZ, with the exception of *PtPIP1;4*, which was expressed preferentially in the DZ (Supplementary Table S4).

### Differential gene expression during the osmotic stress response

**Fig. 4.**
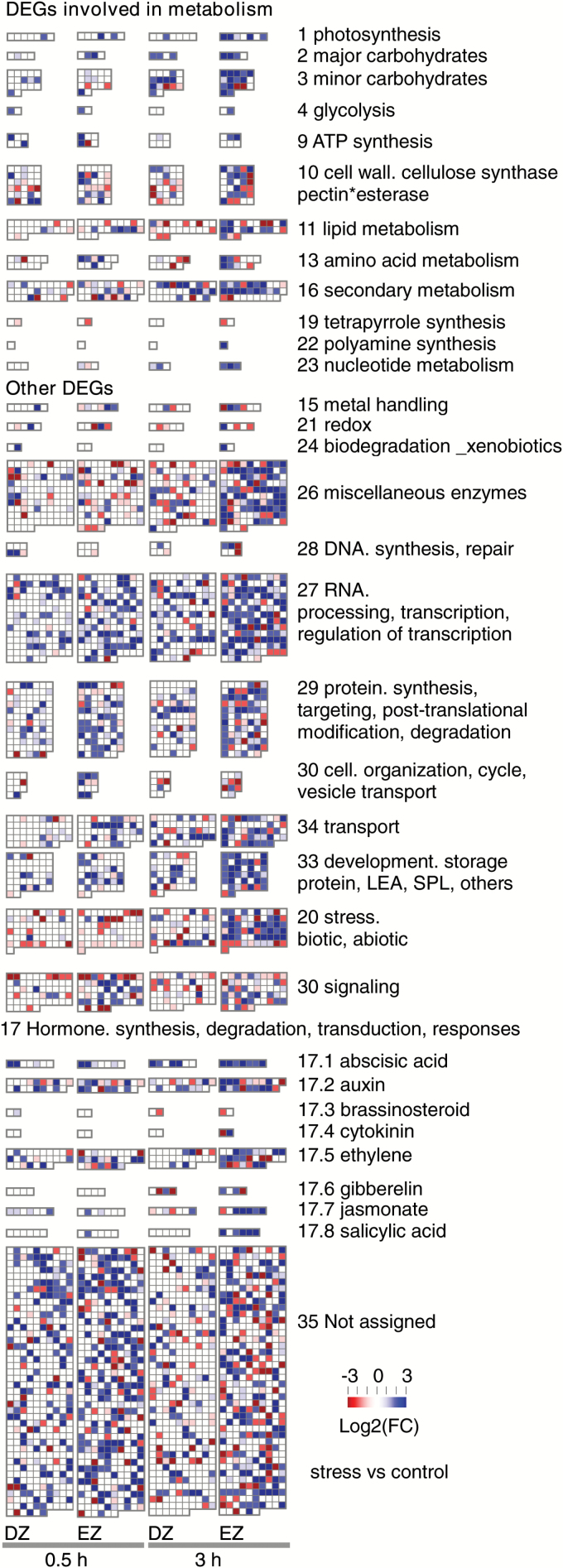
Transcriptional remodeling under osmotic stress. Mapman visualization of PEG-responsive genes retrieved for the division zone (DZ) and the elongation zone (EZ) after 0.5h or 3h under stress (1189 mapped of 1162 data points). Numbers and names refer to bins (http://mapman.gabipd.org/).

Regardless of zone and time, 1162 genes and 53 uTARs were responsive to osmotic stress, with a change in expression above the threshold |Log2(FC)|≥ 2 as compared with the respective control (‘PEG-responsive genes’; Supplementary Tables S3, S4). The stress response covered 26 Mapman bins (Fig. 4; Supplementary Table S4). About one-third of DEGs corresponded to genes with unknown function (bin 35). The stress response covered distinct metabolic pathways (related to cell wall or lipid, amino acid and sugar metabolism, or encoding large enzyme families, such as cytochrome P450) and transporters (including *aquaporin*). However, the key components triggered by osmotic stress were regulatory processes (~330 mapped genes). Several organization levels were involved, as showed by the numerous transcription factors (bin 27.3), as well as genes related to protein modification and degradation (bin 29.4 and 29.5), signaling (bin 30), and hormones (bin 17). Mapping highlighted the fact that the stress-responsive genes were distributed unevenly among the DZ and EZ, the DZ transcriptome being less responsive than the EZ transcriptome. Moreover the number of DEGs increased with time under stress in both zones.

**Fig. 5.**
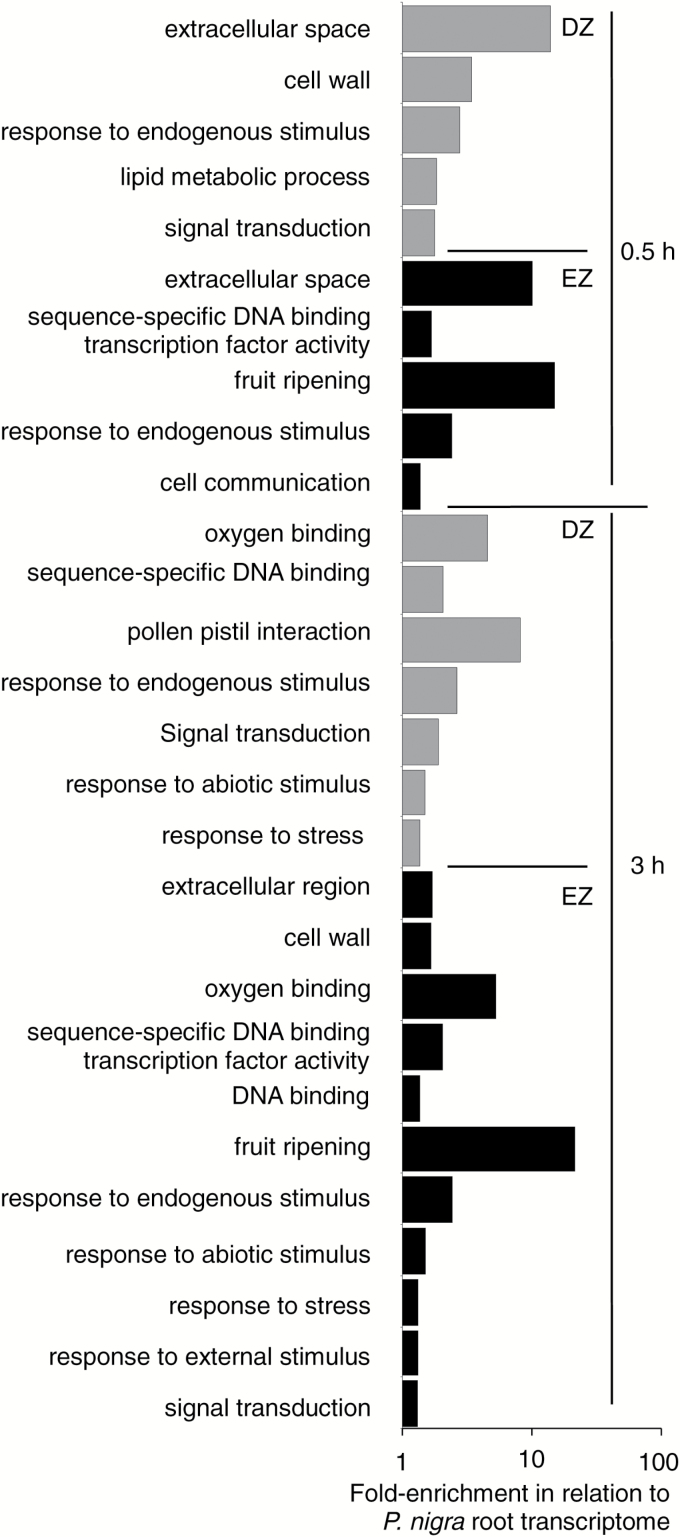
Functional enrichments of the response. Gene ontology fold-enrichments among PEG-responsive genes retrieved for the DZ and EZ after 0.5h or 3h under stress as compared with *P. nigra* root transcriptome. Only non-redundant GO terms are shown (|fold change enrichment| >2, corrected *P*-value <0.05; Supplementary Table S2).

GO analyses were used to test whether the response encompassed functional enrichments. Half an hour after stress onset, 216 DEGs were detected in the DZ (53 down-regulations and 163 up-regulations; Fig. 4), leading to a few functional enrichments (Fig. 5; Supplementary Table S2C). For cellular component, ‘extracellular space’ and ‘cell wall’ were the most enriched terms. The most enriched biological processes were ‘response to endogenous stimulus’, ‘lipid metabolic process’, and ‘signal transduction’, confirming that the osmotic stress was indeed sensed. Meanwhile, 393 DEGs were detected in the EZ (including 302 up-regulations; Fig. 4). Some biological processes were strongly enriched (‘fruit-ripening’, ‘response to endogenous stimulus’, and ‘cell communication’; Fig. 5; Supplementary Table S2D), as well as the molecular function’s ‘sequence-specific DNA binding transcription factor activity’, indicating that signaling and regulatory pathways were activated. The most enriched term for cellular component was ‘extracellular space’ (Fig. 5; Supplementary Table S2D). Three hours after stress onset, enlarged sets of DEGs were retrieved in both the DZ and the EZ (272 and 588 DEGs, respectively), leading to numerous functional enrichments. The temporal deployment of the transcriptional responses was thus converted into physiologically relevant processes. Concerning the DZ (Fig. 5; Supplementary Table S2E), ‘pollen–pistil interaction’, ‘response to abiotic stimulus’, and ‘response to stress’, together with ‘oxygen binding’ and ‘sequence-specific DNA binding transcription factor activity’ were among the most enriched GO terms, whereas they were absent in the early response. For the EZ (Fig. 5; Supplementary Table S2F), enrichments concerned ‘oxygen binding’, ‘DNA binding’, ‘response to abiotic stimulus’, ‘response to stress’, ‘response to external stimulus’, and ‘signal transduction’. For GO terms enriched at both time points, transcriptional responses strengthened with time. Fold-enrichment values were similar or increased, while DEG numbers increased strongly. For instance, the number of DEGs in ‘response to endogenous stimulus’ almost doubled with time.

Our transcriptome analysis revealed that regulations were highly structured by time, highlighting a rapid but labile response (268 DEGs only at 0.5h; Supplementary Table S4). Other DEGs contributed to sustained or late responses, their expression being regulated at 3h. This schedule was consistent with responses to longer stress in poplar and maize apex (Cohen *et al.*, 2010; Opitz *et al.*, 2016; Supplementary Table S4). Using poplar to maize orthology and a stringent threshold for differential expression, a weak overlap was found with maize roots experiencing a 6h long PEG stress (Supplementary Table S4). Most of the overlapping genes were involved in sustained or late responses. In two other poplar species experiencing 36h or 10 d long soil water deprivation (Cohen *et al.*, 2010), most DEGs were common again, with sustained or late responses. Long water deprivation provoked an energy deficit transcriptional response (Cohen *et al.*, 2010), which did not occur here, as expected from a short-term response (Supplementary Table S4). Species, growth conditions, plant stature, stress application and duration, and even internal cell status distinguished the poplar studies, and 190 highly responsive genes constituted an unexpectedly high overlap. For ~80% of them, the direction of transcriptional regulation was identical, suggesting their key roles for stress acclimation. DEGs strongly responsive to both PEG and drought might be constitutive components of the water deficit response in poplar roots.

## Discussion

Plant growth is intimately intertwined with environmental conditions, and growth plasticity is a crucial property that allows sessile organisms to withstand stress. Water distribution in soil, as well as water availability, fluctuate in both time and space. Local water depletion acts both as a stress and as a positional cue shaping root systems (Robbins and Dinneny, 2015). Elucidating how changes in water availability are transmitted to changes in root growth remains a current focus. In the root axis, cells coming from the DZ rapidly enlarged within the EZ, making the root apex relevant to dissecting the cellular components of stress-inducible responses (Baskin, 2013). The spatial patterning of these responses has been documented at several scales, increasing our knowledge of the complexity of controls underlying root growth variation (reviewed in Yamaguchi and Sharp, 2010). While most of these studies focused on responses in the mid-term, here we documented the earliest impacts of osmotic stress on the growing root of *Populus*, focusing on the dynamics of cell processes and the establishment of underlying controls.

### Kinetics and kinematics reveal the sensitivity of cell division and cell elongation in *Populus* root

Following osmotic stress onset, root growth was strongly reduced but recovered rapidly (Fig. 1). After some oscillation, the growth rate stabilized at a lower level than in controls. While not mentioned by the few studies that have monitored growth following osmotic stress onset (Pritchard *et al.*, 1991; Frensch and Hsiao, 1994), such oscillations could result from the different time courses of the processes underlying growth recovery, such as osmoregulation, cell wall relaxation, or water transport. Osmotic stress shortened the growth zone and reduced the cell elongation rate (Fig. 1), as previously shown for other species (Sharp *et al.*, 1988; Pritchard *et al.*, 1991; Triboulot *et al.*, 1995; Sacks *et al.*, 1997). A paradigm arose from these latter studies, stating that cell elongation is insensitive to water stress in the apical part of the root. In contrast, we recently showed that stress-driven impairment of cell elongation encompassed this apical region (corresponding to the DZ) in a euramerican poplar hybrid (Bizet *et al.*, 2015*b*). Consistently, *P. nigra* exhibited a significant reduction in cell expansion rate within the DZ, indicating that cytoplasmic growth was also impacted by osmotic stress. In maize, water stress caused a large change in the cell production rate and local cell division rate profile (Fraser *et al.*, 1990; Sacks *et al.*, 1997). Here, osmotic stress rapidly impaired the cell production rate without affecting DZ length (Fig. 1). Since stress duration was very short compared with the ~20h cell cycle (Ivanov, 1997; Bizet *et al.*, 2015*b*), a shortening of the DZ would have required numerous precocious exits of the cell cycle. Similarly, 10h of osmotic stress was too short to induce mitotic exit in Arabidopsis leaves (Skirycz *et al.*, 2011).

Stress-induced transcriptional remodeling was surveyed separately in the DZ and EZ. The DZ encompasses dividing and slowly expanding cells, while the EZ has rapidly elongating cells (Supplementary Fig. S2). The EZ was shortened but the sampling size was kept identical regardless of growing conditions. Stressed EZ samples thus included a small quantity of mature tissue, but contamination was negligible since RNA concentration decreases strongly moving away from the quiescent center as a result of dilution by cell expansion (Merret *et al.*, 2010). Functional enrichments, clustering, and cross-comparison with gene profiling in Arabidopsis confirmed that zones were properly separated from each other. The DZ transcriptome was consistent with dividing and slowly expanding cells, while the EZ transcriptome was associated with rapidly elongating cells.

### The build-up of hormonal control underlying growth inhibition

**Fig. 6.**
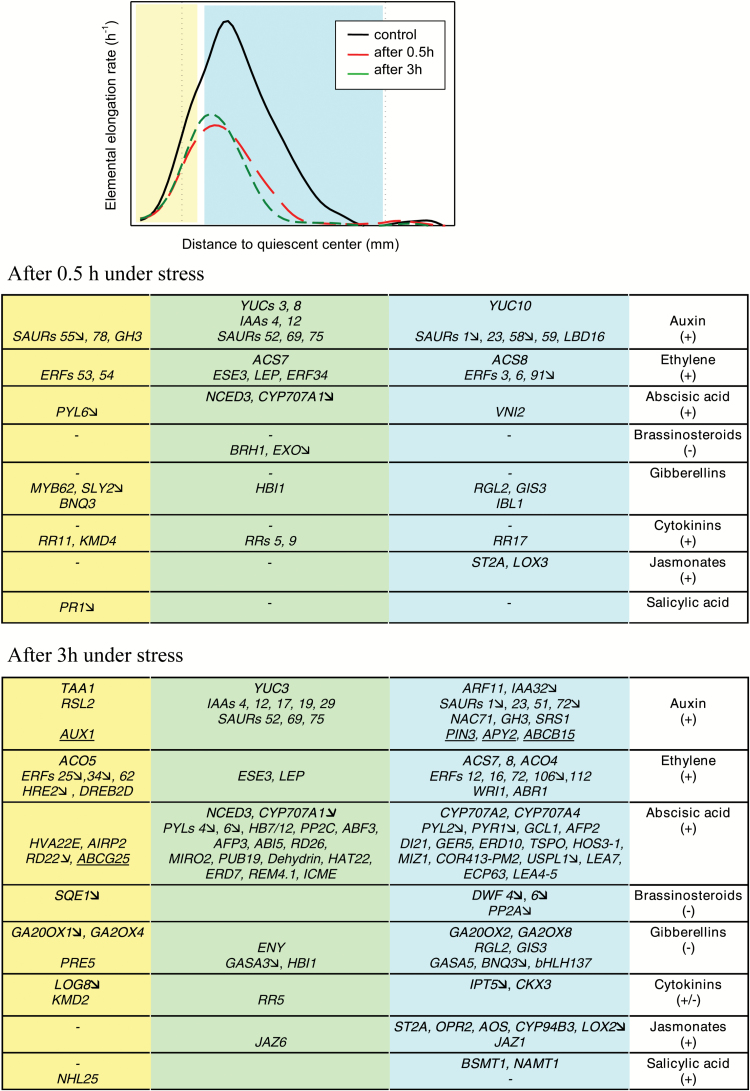
Hormone responses during osmotic stress root growth inhibition. The growth response is summarized by typical profiles of the elemental elongation rate before stress, and after 0.5h and 3h under stress. Tables summarized PEG-responsive genes related to hormone metabolism and signaling, as well as hormone-responsive genes (Supplementary Table S4). Down-regulated expression was indicated with an arrow. Metabolic genes are in bold and hormone carriers are underlined. Yellow, blue, and green backgrounds corresponded to the spatial patterns of gene regulation (DZ, EZ, and both zones, respectively).

Our experimental set-up combined a proper application of osmotic stress—that controls for hypoxia and prevents root manipulation—with a sampling strategy based on fine analysis of root growth. In this context, gene expression profiling was a powerful tool to reveal physiologically relevant processes with regards to growth inhibition. Depicting the early molecular events triggered by osmotic stress, we showed that genes related to hormones (including metabolism, primary targets, and signaling cascades) encompassed 10% of DEGs after 0.5h under stress, and up to 20% after 3h. Hormone networks have been dissected in *A. thaliana* roots (Takatsuka and Umeda, 2014; De Smet *et al.*, 2015). Meanwhile most knowledge about hormonal control of root growth has been generated from artificial modulation of their levels—by hormone applications and/or targeted mutants. Here, the build-up of hormonal interplays that accompanied root growth inhibition was dissected under physiological conditions and is summarized in Fig. 6.

Ethylene and auxin pathways were rapidly elicited (Fig. 6). The expression of auxin biosynthetic genes increased (flavin monooxygenase, *YUCCAs* and the tryptophan aminotransferase gene, *TAA1*), suggesting *de novo* auxin biosynthesis. Consistently, auxin signaling and response were activated (*AUX/IAA*, *GH3*, *LBD16*, *SAUR*; Fig. 6; Okushima *et al.*, 2005). Cell-to-cell transport of auxin is mediated by efflux and influx carriers (ABCB, PIN, and AUX families) and influenced by APYRASEs (Kaneda *et al.*, 2011, Liu *et al.*, 2012). A localized activation of these genes occurred after 3h under stress (Fig. 6), in accordance with their drought responsiveness in other species (*OsPin3t*, Zhang *et al.*, 2012; *APY2* and *ABCB15*, Cohen *et al.*, 2010). Local auxin maxima and auxin gradient might thus be reshaped along stressed roots. Transcript accumulation of *1-Amino-Cyclopropane-1-Carboxylate Synthase and Oxidase and Ethylene Response Factor* supported stress-driven accumulation of ethylene (Vandenbussche *et al.*, 2012). Together, our results fit a model of ethylene-mediated inhibition of root growth by a modulation of auxin biosynthesis and transport.

As classically reported under osmotic stress, the ABA pathway was activated (Fig. 6). In Arabidopsis, AtNCED3 controls the level of endogenous ABA under drought (Iuchi *et al.*, 2001). Here, sustained activation of poplar orthologs suggested an enhanced ABA biosynthesis together with a decreased catabolism (*ABA 8'-hydroxylase* genes, *CYP707A1*). During the early response, ABA increase was supported by transcriptional regulation of an ABA receptor (*PYL6*) and an ABA-dependent transcriptional activator (*VND-INTERACTING2*; Yang *et al.*, 2011). However, key ABA-responsive genes were not yet activated, indicating that signaling was only primed. Among the ABA-dependent pathway (Finkelstein, 2013), *ABI5*, *AFP*, *ABF*, and *RD26* were activated after 3h under stress. Additionally, *PYL* expression was repressed while expression of *PP2C* genes and *HB7/12* was increased (Fig. 6). In Arabidopsis, *ATHB12* and *ATHB7* transcription factor genes mediated a negative feedback by up-regulating the expression of *PP2C* and down-regulating the expression of *PYL* (Valdès *et al.*, 2012). An ABA exporter-encoding gene, localized in the plasma membrane in Arabidopsis (*ABCG25*; Kuromori *et al.*, 2010), was transcriptionally activated in the DZ. As in droughted roots (Cohen *et al.*, 2010), genes related to ABA catabolism (*CYP707A2* and *CYP707A4*) were activated in the EZ. Together, ABA appeared finely tuned in stressed roots via metabolic and transport feedback. ABA-mediated transcriptional responses occurred in both zones, but required some time to establish fully. Such dynamics support the canonical role of ABA accumulation under drought, counteracting the growth inhibition due to ethylene accumulation (Sharp, 2002).

Levels of the growth-promoting hormones brassinosteroids and gibberellins were decreased after 3h under stress (Fig. 6). Among brassinosteroid primary targets (Coll-Garcia *et al.*, 2004), expression of *BRH1* and *EXO* genes suggests that brassinosteroids were lowered rapidly (Fig. 6). The transient repression of several *EXO* genes drove functional enrichments in ‘extracellular space’ in both zones (Fig. 5). Beyond being a hallmark of brassinosteroid status, *EXO* was proposed as a potential mediator of the brassinosteroid-promoted growth necessary for cell expansion (Schröder *et al.*, 2009; Aya *et al.*, 2014). Later, repression of brassinosteroid biosynthetic gene expression (*SQE1* and *DWF* genes) occurred in a zone-preferred manner (Fig. 6). *DWF4* repression could reflect negative feedback (Zhang *et al.*, 2009), but the decrease in brassinosteroids was confirmed by *PP2A* repression (Tang *et al.*, 2011). Gibberellins have been proposed to mediate growth inhibition and physiological responses to drought (Zawaski and Busov, 2014). Here, early responsive DEGs included transcriptional factors (*MYB62* and *GIS3*), DELLA protein (*RGL2*), and F-Box protein (*SLY2*), which are all hallmarks of a decrease in gibberellin. However, the regulation of *bHLH* transcription factor (*bHLH137*, *BNQ3*, *PRE5*, *HBI1*, and *IBL1*) involved in gibberellin and brassinosteroid signaling pathways (Ikeda *et al.*, 2012) precluded any firm conclusions. Homeostasis of bioactive gibberellins results from their production via the action of GA-20-oxidases, and their inactivation by catabolic GA-2-oxidases (Hauvermale *et al.*, 2012). Thus, transcriptional regulation of *GA2OX4*, *GA2OX8*, and *GA20OX1* was expected to reduce gibberellin (Fig. 6). The signaling cascade, responses, and feedback were consistent with lowered gibberellin levels (Zhang *et al.*, 2009; Feurtado *et al.*, 2011). ABA antagonized gibberellin and brassinosteroid pathways (Zentella *et al.*, 2007; Zhang *et al.*, 2009). Consistent with our data, ATHB12 was reported to decrease *GA20OX1* expression and growth (Son *et al.*, 2010). Overall, growth-promoting hormones were reduced consistently with stress inhibition of root growth.

Artificial increases in cytokinins negatively impacted root growth and DZ length in Arabidopsis (Werner *et al.*, 2003). Cytokinin signals depend on its transmission through a multistep phosphorelay [RESPONSE REGULATORS (RRs) Müller, 2011] and its modulation by KISS ME DEADLY F-box proteins (H.J. Kim *et al.*, 2013). As shown in Fig. 6, accumulation of *RR* transcripts suggested that cytokinin levels increased in stressed roots. Bioactive cytokinins depend on *de novo* synthesis (IPTs, isopentenyl transferases), catabolism (CKX, cytokinin oxidase/dehydrogenase), and activation (LOG, lonely guy) (Kuroha *et al.*, 2009). In Arabidopsis, dehydration or ABA treatments reduced *IPT* expression but also, as a secondary response, repressed *CKX* expression (Nishiyama *et al.*, 2011). Here, osmotic stress repressed *IPT5* expression while increasing *CKX3* expression in the EZ. These responses mimicked the negative feedback provoked by cytokinin treatment (Brenner and Schmülling, 2012). In the DZ, the levels of bioactive cytokinins appeared tuned through regulatory rather than metabolic processes (*LOG* and *KMD*; Fig. 6). Alteration of cytokinin status and signaling thus plays a role in the inhibition of root growth.

Defense hormones also participate in stress responses (Fig. 6). Osmotic stress provoked a rapid activation of jasmonate metabolism in the EZ. *ST2A*, *LOX2*, and *OPR2* were drought responsive in poplar roots (Cohen *et al.*, 2010), and *LOX5* and *OPR3* in the EZ of maize roots (Opitz *et al.*, 2016). Jasmonates were then perceived and *JAZ* activated. Consistent with our results, jasmonates inhibited root growth through interaction with auxin (Chen *et al.*, 2011). Salicylic acid (SA) fluctuated during the stress response (Figs 4–6). In the DZ, early repression of SA-inducible *PR1* was followed by the up-regulation of SA-inducible *NHL25*. In the EZ, the pathway of SA conversion into volatile methylsalicylate was activated, suggesting a systemic signaling (Chen *et al.*, 2003).

Distinct sets of genes were recruited in the DZ and the EZ (Fig. 6). Osmotic stress reshaped hormonal status and probably modified local hormone maxima, controlling cell proliferation and expansion.

### Impairment of growth machinery

In addition to changes in hormonal status, growth response was accompanied by a strong remodeling of transcription factors, kinases, and regulators of protein turnover, lipid metabolism, cell wall properties, and transporters (Fig. 4). Growth was restricted, but stress promoted the expression of growth effectors. As found in maize (Yamaguchi and Sharp, 2010; Opitz *et al.*, 2016), transcriptional regulation occurred for genes related to cell wall properties, including *EXPANSIN*, *XTH*, *PECTIN LYASE*, *PECTIN METHYL ESTERASE*, and *CELLULOSE SYNTHASE* (Cosgrove, 2005). The cell wall-strengthening *XTHs* were down-regulated, and the wall-loosening *EXPs* (especially *EXPA1* and *EXPA8*) and *PECTINE LYASE* genes were up-regulated. In the root of stressed maize, the pattern of *EXPA1* accumulation fitted expansion maintenance, contributing to growth maintenance of turgor-reduced cells (Wu *et al.*, 2001). Its ortholog accumulated over the whole poplar growth zone, but the cell expansion rate was not maintained, suggesting that facilitation of expansion via *EXPA* expression might not be sufficient to counteract stress impact. Osmotic stress also elicited expression of *aquaporin*, *PtPIP2;7*, *PtPIP2;10*, *PtTIP1;1*, *PtTIP1;2*, *PtTIP2;2*, *PtTIP2;3*, and *PtTIP2;4* (Supplementary Table S4). Although members of this multigene family show redundancy and high stress responsiveness (Cohen *et al.*, 2013), these transcriptional regulations suggested an increase in membrane hydraulic conductivity (Maurel, 2007). As cell expansion rate depends on wall extensibility as well as on membrane hydraulic conductivity (Lockhart, 1965), the up-regulation of *aquaporin* also pointed to facilitation of cell expansion.

The cell production rate was reduced by osmotic stress but none of the core cell cycle genes was retrieved among the most stress-responsive genes in the DZ, even if they were detected as preferentially DZ-expressed. In this short-term response, post-transcriptional regulation of cell cycle components could be responsible for the reduction of the cell division rate, as demonstrated in Arabidopsis leaves (Skirycz *et al.*, 2011). On the other hand, the reduced growth rate may underlie this response. Cells have to reach a size threshold before dividing, so lowered cytoplasmic growth may participate in control of the cell cycle, as previously proposed (Bizet *et al.*, 2015*b*). These working hypotheses are not exclusive and both require further examination.

RNA-sequencing provided a genome-wide diagnostic of the molecular regulation underlying stress responses. Many DEGs were also drought-responsive genes that contributed to acclimation to soil water deprivation (Cohen *et al.*, 2010), suggesting that drought and osmotic stress, as well as short- and long-term responses, share common components. In conclusion, our work revealed that osmotic stress interfered with growth by changing hormonal status and activating regulatory proteins rather than by repressing the underlying machinery of expansive growth.

## Supplementary data

Supplementary data are available at *JXB* online.

Table S1. Number of sequenced and single-mapped reads in the 32 libraries.

Table S2. Gene ontology analyses reduced to most specific terms.

Table S3. *In silico* validation of unannotated transcriptionally active regions (uTARs).

Table S4. Differentially expressed genes.

Figure S1. Osmotic pressure and oxygen saturation of nutrient solutions.

Figure S2. Growth traits within *P. nigra* root.

Figure S3. qPCR validation: primer sequences and expression patterns.

Supplementary Data

## Abbreviations:

DEG: differentially expressed gene
EER: elemental elongation rate
uTAR: unannotated transcriptionally active region.
