# Supplementary material for: The build-up of osmotic stress responses within the growing root apex using kinematics and RNA-sequencing
Source: J Exp Bot. 2016 Oct 4;67(21):5961–73. doi: 10.1093/jxb/erw350 (PMC5100013; doi:10.1093/jxb/erw350)
Supplement: Supplementary Data [file supp_67_21_5961__index.html]

The build-up of osmotic stress responses within the growing root apex using kinematics and RNA-sequencing — The build-up of osmotic stress responses within the growing root apex using kinematics and RNA-sequencing — Supplementary Data 

# The build-up of osmotic stress responses within the growing root apex using kinematics and RNA-sequencing

## Supplementary Data

Data files

- supplementary\_figures\_S1\_S3.pdf - Supplementary Data
- supplementary\_table\_S1.xls - Supplementary Data
- supplementary\_table\_S2.xls - Supplementary Data
- supplementary\_table\_S3.xls - Supplementary Data
- supplementary\_table\_S4.xls - Supplementary Data
