# Supplementary material for: The build-up of osmotic stress responses within the growing root apex using kinematics and RNA-sequencing
Source: J Exp Bot. 2016 Oct 4;67(21):5961–73. doi: 10.1093/jxb/erw350 (PMC5100013; doi:10.1093/jxb/erw350)
Supplement: Supplementary Data [file supp_erw350_supplementary_figures_S1_S3.pdf]

Fig. S1: Osmotic pressure (A) and oxygen saturation (B) of nutrient solutions. Time=0 corresponds to the stress onset. The osmotic potential of a solution equals the opposite of its osmotic pressure

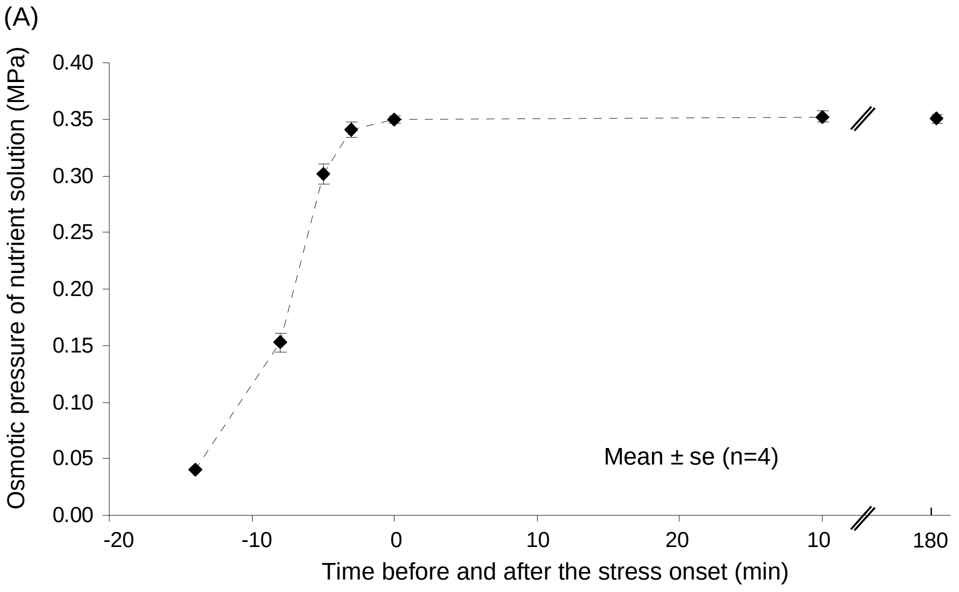

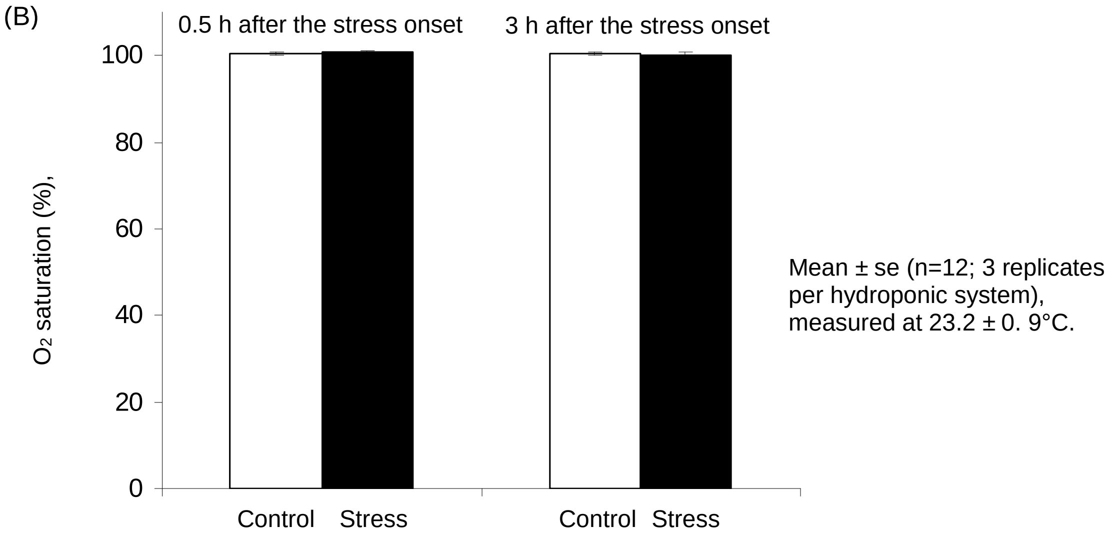

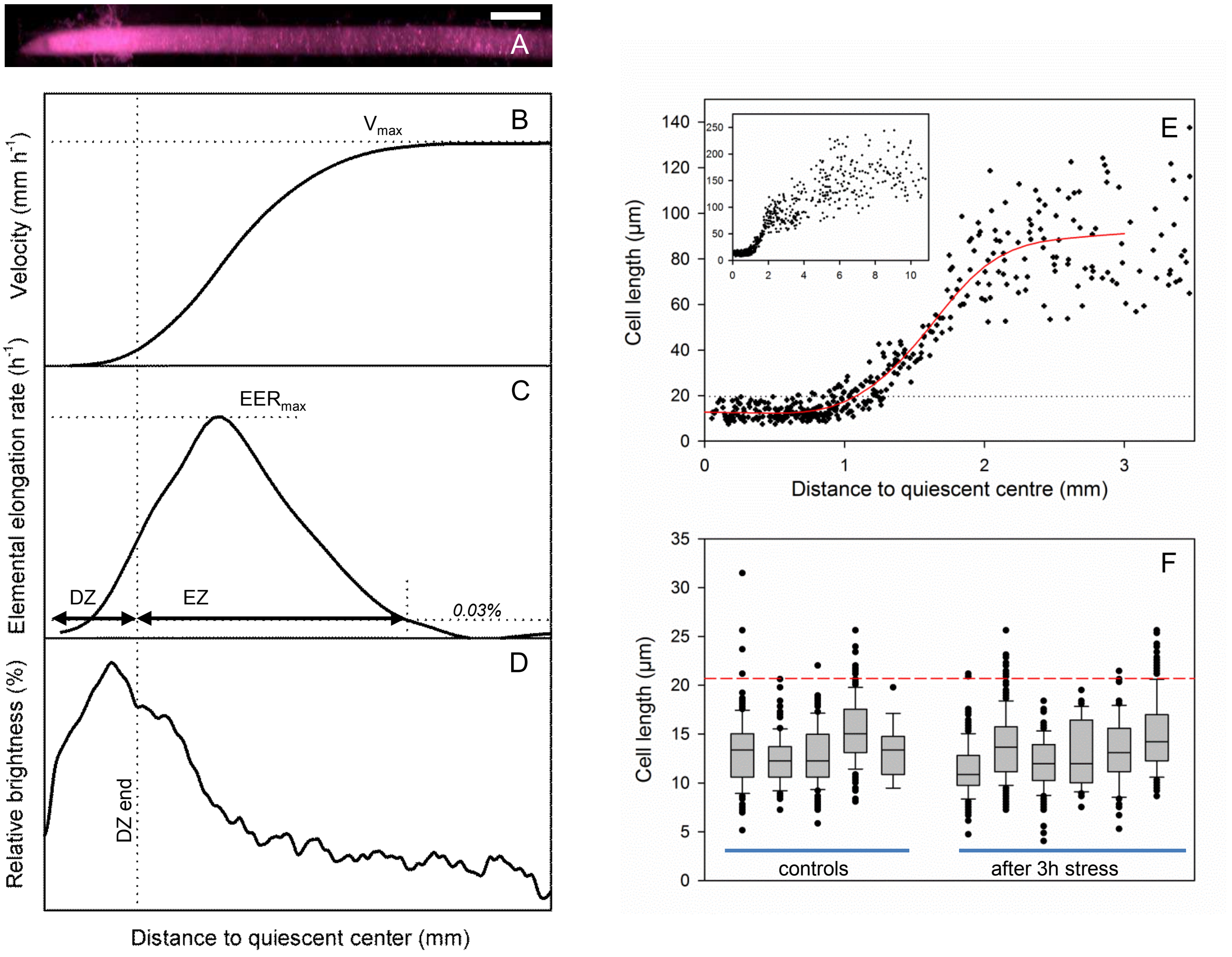

Fig. S2: Growth traits within *P. nigra* root.

(A) Root apex taken under infrared light, used for kinematics and determination of division zone length. Scale bar = 1 mm.

(B, C, D) Typical profile of velocity, elemental elongation rate and brightness along the root apex, respectively. V_{max}: maximum velocity. EER_{max}: maximum elemental elongation rate. DZ end: limit of the division zone (DZ). EZ: elongation zone.

(E) Typical cell length profile in the first millimeters of the root apex. The red line corresponds to the smoothspline . Inside graphe : Typical cell length profile along the whole root apex.

(F) Cell length distribution within the first 500 µm of the meristem, in 5 to 6 individual roots, for each treatment. The dotted red line corresponds to the length threshold separating small and proliferative cells from large fast-expanding cells. Length of 99% of cortical cells within the apical part of the cell proliferation domain were below 20.7 µm (see Bizet et al, 2015 for more details).

Fig.S3: QPCR validation: primer sequences and expression patterns

a) Primer sequences. 19 genes and 4 unannotated transcriptionally active regions were selected for validation. Five genes and the external control (Alien QRT-PCR Inhibitor Alert kit, Stratagene, TX, USA) were the most stable genes and used as reference (geNORM v3.5). All amplicons were sequenced.

Gene model v3.0	Symbol in figure S2b	Forward sequence 5' to 3'	Reverse sequence 3' to 5'	Previously published in
**reference genes**				
Potri.010G127500		CATGTTTTGCTCCCACCTCT	CCGCAACTCTGTCTTCAACA	Cohen et al., 2010
Potri.001G287100		GCTGATGGATCCAAAGTTCA	GCGGAAAATGAAGGATTGAC	
Potri.002G105100		CCTTACGTGCAAAGCTGACA	AGGAATTGGCCTGTGTAAC	
Potri.012G071600		TAATCATCGTGGCAGCCAAT	GGCAACAGATGACATGAAAC	Dumont et al., 2014
Potri.004G133500		GTTTGGAGCAGGAAGACGAG	CTCACTGTGCACCACATCCT	
**gene of interest**				
Potri.016G125400	*PYL4*	GTTGTTGGTGGGGACCATA	ACCGCGTAAGACTCCATGA	Bizet et al., 2015a
Potri.001G393800	*NCED3.1*	AAGACCCGGTTCGCTTACTT	CTCCGTAAGTGTGCTTGTGT	
Potri.011G112400	*NCED3.2*	GAAAATCCCGGTTTGCTTACT	ACAAAGGCTCTCCACCGAAT	
Potri.009G027200	*TIP1.2*	GGGTGACATCGGGATAATTG	TGCAGAACCAACGAATGGAC	Cohen et al., 2010
Potri.008G050700	*TIP1.4*	AATCCTGCTGTGACCTTTGG	GAAGCAAGCAAGCAACAACA	
Potri.003G050900	*TIP2.2*	ATTTTCTGGTGGGTCCATGA	TAAGTCCAGCTAGCCCTCCA	
Potri.008G057100	*EXPA7*	CGGTGTTTACACTGGTGCAG	CAAGTTACCGTACCCGCAAG	
Potri.015G070300	*E2F*	TGAGAAGACCCACACTGCTG	CTCTCTCGGCGACAGTTACC	
Potri.016G045500	*ACT1.1*	CACACTGGAGTGATGGTTGG	ATTGGCCTTGGGGTTAAGAG	Merret et al., 2010
Potri.010G191000	*AUX1*	TCAGCTCGACAGAATGCAGT	TCCCAATCCAAAGCCAACCA	
Potri.005G163300	*EXO*	TGGCTATTTCCAGGGTCCAA	AATCCCCAGCATAACCAGGA	
Potri.001G048200	*ESE3*	GAAACCGGCTCAAAATGGCA	ACTCCATGGACCCACAATCT	
Potri.014G117300	*GA2OX4*	TGACCATCTTGCGATCCAAC	GGAATCACCCACAATCACGT	
Potri.014G120000	*RGL2*	CAATTTGACCGTGCAAGCAG	TTTCTGGTGGACGCTAAGGT	
Potri.008G202200	*IPT5*	TGTATGCTAGCTCGTCGACA	TGTCAGCCTCCTTTCCACTT	
Potri.015G070000	*RR5*	TTTCTGCCGTCCGAACTTCA	ACGGTTTCCCTACACTTGCT	
Potri.006G139400	*JAZ1*	GGGCTCCTCGGAATTTTACA	TTGTCAGCCGGGAAATCGTT	
Potri.001G167700	*LOX3*	AGAGGTGTCCCAAATAGCGT	CCAACTTTGAGCGGCCTAAA	
Potri.019G022600	*BSMT1*	TTCCTCTTGCAGCCTACACT	GACTTGCCTTCGCCATGTAT	
**uTARs (position on *P. trichocarpa* genome V3.0)**				
Chr01:6487015..6489547	*uTAR1*	CAACTGCCACCACCGAAAAT	AGACGAGAACTGAACGAGGA	
Chr01:28461864..28464288	*uTAR2*	ACCAGCTTGCTTCCCAAGAA	AGGGGCCAATTCCAAGGTTT	
Chr04:5084287..5085477	*uTAR3*	TGGCAATCGGTTAGTTGGGT	CGGACTGCCTTGGTCATTAT	
Chr07:12386793..12388104	*uTAR4*	TCACCAAACCTAGTCGCAGA	TCCATGCGTTGATGTGCTGA	

B) Fold changes in gene expression assessed by RNA-Sequencing (in black, mean values computed from normalised FPKM values- only significant regulation are shown, corrected P-value<0.05) and qPCR (in grey, mean ± standard error of the ratio). qPCR were performed when a significant regulation was detected by RNA-sequencing.

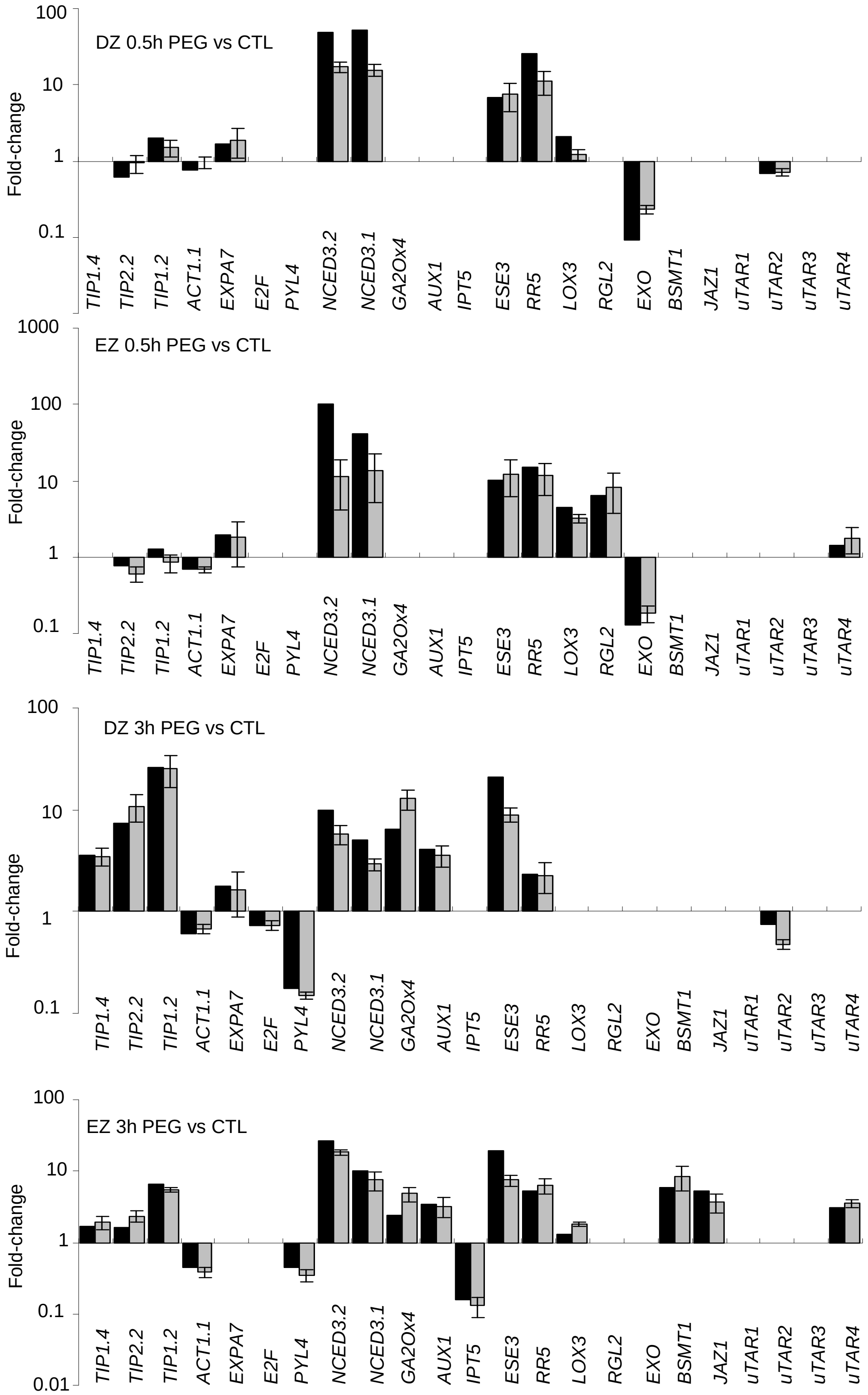

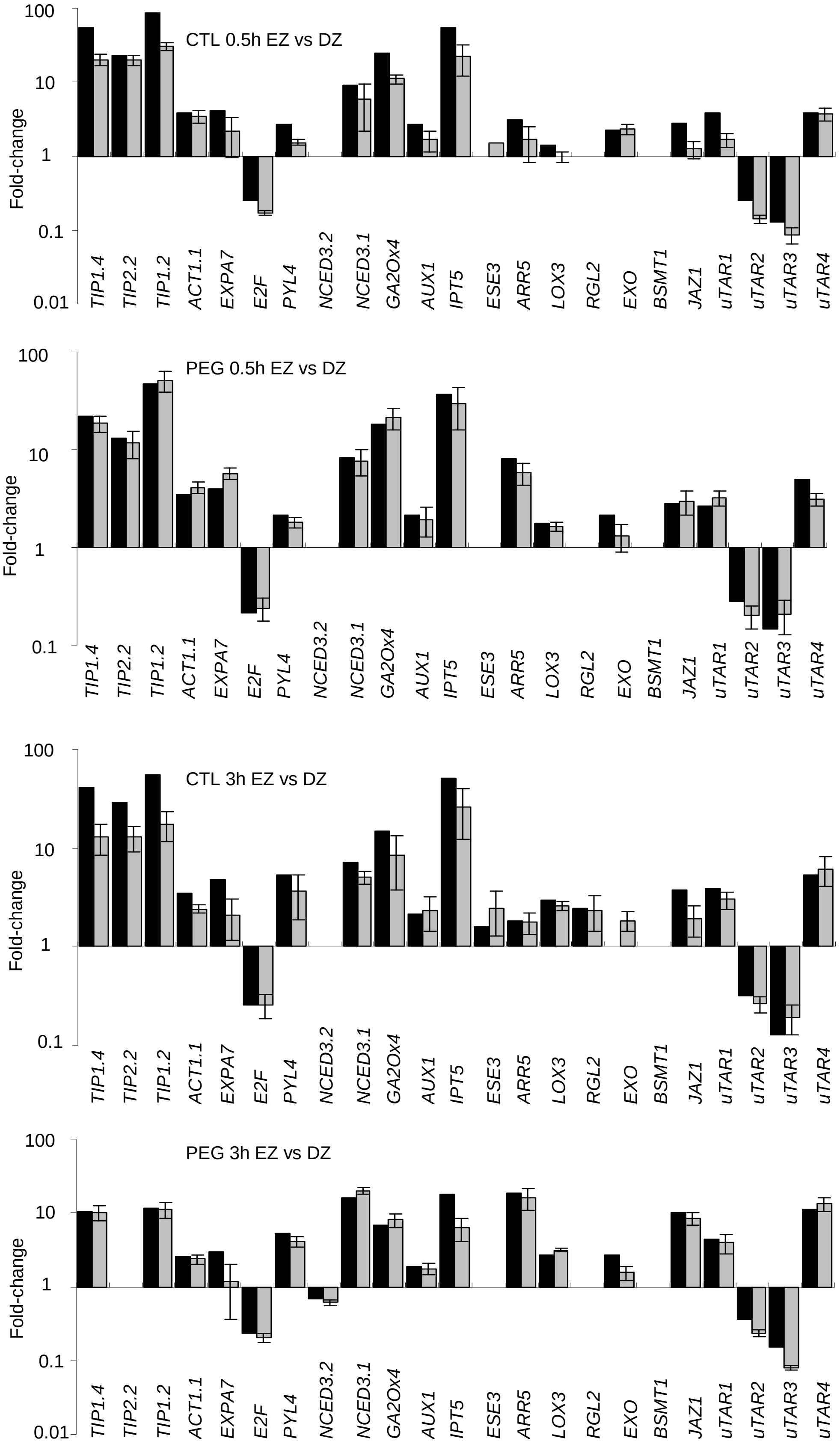
CTL 0.5h EZ vs DZ
PEG 0.5h EZ vs DZ
CTL 3h EZ vs DZ
PEG 3h EZ vs DZ
Fold-change
100
10
1
0.1
0.01
TIP1.4
TIP2.2
TIP1.2
ACT1.1
EXPA7
E2F
PYL4
NCED3.2
NCED3.1
GA2Ox4
AUX1
IPT5
ESE3
ARR5
LOX3
RGL2
EXO
BSMT1
JAZ1
uTAR1
uTAR2
uTAR3
uTAR4
